# Co-occurring DMD, GJA1, and novel FYCO1 variants in a proband from a consanguineous oculodentodigital dysplasia family: a rare multi-locus case report

**DOI:** 10.3389/fgene.2026.1753212

**Published:** 2026-03-04

**Authors:** Kondyarpu Abhishek, Nitish Kumar Mohanta, Joseph John, Swarupa Panda, Amit Kumar Satapathy, Roma Rattan, Puppala Venkat Ramchander

**Affiliations:** 1 Institute of Life Sciences, Bhubaneswar, India; 2 Department of Pediatrics, All India Institute of Medical Sciences, Bhubaneswar, India; 3 Department of Pediatrics, Shrirama Chandra Bhanja (SCB) Medical College and Hospital, Cuttack, India; 4 Department of Biochemistry, Shrirama Chandra Bhanja (SCB) Medical College and Hospital, Cuttack, India

**Keywords:** consanguinity, DMD, duchenne muscular dystrophy, FYCO1, GJA1, multi-locus inheritance, oculodentodigital dysplasia, trio exome sequencing

## Abstract

Whole-exome sequencing of the proband and the family revealed multi-locus pathogenic variants (MGVs) leading to multiple genetic diagnoses (MGDs), explaining the complex phenotype with neuromuscular, ocular, and craniofacial abnormalities. The proband harbored a *de novo* hemizygous *DMD* frameshift variant consistent with Duchenne muscular dystrophy, a paternally inherited heterozygous *GJA1* in-frame indel associated with oculodentodigital dysplasia (ODDD), and a novel homozygous *FYCO1* nonsense variant causing congenital cataract. Fraction of ROH (FROH) analyses indicated extended autozygosity, which is indicative of second-cousin-level consanguinity. The novel *FYCO1* variant was located within one of the indicative ROHs, supporting identity by descent. Structural analysis predicted truncating or domain-disrupting effects across all three genes, aligning with the multisystem phenotype. The coexistence of the *DMD*, *GJA1*, and novel *FYCO1* variants in a single individual is exceptionally rare. To our knowledge, this represents the first report of such a multi-locus combination, highlighting the diagnostic complexity of combined recessive, dominant, and *de novo* events in a proband born in a consanguineous ODDD family.

## Introduction

Next-generation sequencing (NGS), particularly whole-exome sequencing (WES) and whole-genome sequencing (WGS), has revolutionized the diagnosis of rare Mendelian disorders by uncovering novel molecular mechanisms and broadening the recognized phenotypic spectra (Baldridge et al., 2017). These advances have also highlighted multi-locus disease-causing genomic variations (MGVs) and multiple genetic diagnoses (MGDs), in which pathogenic variants at two or more independent loci give rise to blended clinical features (Posey et al., 2017). Such findings complicate diagnosis, management, and genetic counseling, as each condition carries distinct inheritance patterns and recurrence risks. The growing recognition of MGVs and MGDs marks a paradigm shift in Mendelian genetics and underscores the need for clinical geneticists to integrate molecular and bioinformatics expertise with traditional evaluation for accurate phenotype–genotype interpretation (Hennekam and Biesecker, 2012).

Through the lens of WES and WGS, MGDs have been detected in 1.4%–7.2% of cases, challenging the traditional single-diagnosis paradigm and highlighting the need for more refined, multilayered clinical approaches (Narayanan et al., 2021; Smith et al., 2019). In this context, trio-based whole-exome sequencing (trio-WES) has been shown to yield higher diagnostic rates than proband-only or traditional methods, further enhancing accuracy while highlighting the need for additional studies to evaluate its clinical utility and economic impact (Lee et al., 2014).

In this study, we report a proband with Duchenne muscular dystrophy (DMD) and congenital bilateral cataracts from a consanguineous family affected by oculodentodigital dysplasia (ODDD). Trio-based exome sequencing and clinical correlation revealed disease-causing variants at multiple loci, with additional phenotypes. These findings provide important insights for therapeutic planning and recurrence-risk counseling for the family.

DMD (OMIM 310200) is an X-linked disorder marked by progressive muscle weakness and wasting. Globally, it affects an estimated 7.1 per 100,000 male individuals (2.8 per 100,000 in the general population), with a birth prevalence of 19.8 per 100,000 live male births (Crisafulli et al., 2020). The DMD gene is the largest in the human genome; due to its size (∼2.5 Mb, 79 exons), it is prone to various mutations (Muntoni et al., 2003; Aartsma-Rus et al., 2016). The reported variants consist of large deletions, duplications, insertions, and point mutations, with nearly one-third occurring *de novo* (Aartsma-Rus et al., 2006; Gibbs et al., 2019; Bello et al., 2016; Zambon et al., 2022; Torella et al., 2020). There were 80% big rearrangements (69% deletions and 11% duplications) and 20% small variants (25% small deletions, 9% insertions, 14% splice-site, and 52% point mutations—mostly nonsense) among the 7,149 DMD mutations found in the TREAT-NMD database (Bladen et al., 2015). Individuals harboring these genetic defects typically exhibit progressive degeneration of skeletal, respiratory, and cardiac muscles, ultimately resulting in premature death (Birnkrant et al., 2018).

ODDD (OMIM 164200) is an extremely rare inherited disorder that develops due to pathogenic variations in the GJA1 gene, which codes the gap junction protein connexin 43 (Paznekas et al., 2003). A wide range of neurological and physical disorders are the outcome of the syndrome’s high penetrance and diverse clinical manifestations (Judisch et al., 1979). The defining skeletal characteristic is bilateral syndactyly of the fourth and fifth digits, which is often associated with camptodactyly, clinodactyly, and toe syndactyly. Craniofacial features consist of a slender nose with underdeveloped alae nasi, microcephaly, anteverted nares, and a large columella, while the hair and nails may exhibit thinness, sparsity, and brittleness (Reisner et al., 1969). Common signs of dental abnormalities include microdontia, enamel hypoplasia, caries, and early tooth loss (Hindu and Umer, 2023). The ocular phenotype may encompass microphthalmia, microcornea, iris abnormalities, cataracts, glaucoma, and optic atrophy (Kumar et al., 2020). Moreover, neurological problems may manifest, such as spastic paraparesis, ataxia, dysarthria, and neurogenic bladder, with certain individuals displaying mild intellectual disability. ODDD manifests as a multisystem illness characterized by significant phenotypic diversity, even among the affected individuals within the same family.

Congenital cataract is a rare yet significant cause of childhood blindness, occurring in 0.6–9.3 cases per 10,000 live births (Shee et al., 2016). It is the consequence of developmental defects or genetic mutations that result in a reduction in lens transparency. Congenital (within 1 year) and juvenile (within 10 years) forms are present; they are frequently isolated but may occasionally be syndromic with ocular or systemic anomalies (Chan et al., 2012). Diagnoses and treatments have been enhanced by advancements in pediatric surgery, imaging, and DNA sequencing technologies (Gillespie et al., 2016). Although amblyopia treatment and prompt intervention can help manage glaucoma and impaired vision, early surgery is essential for improved visual outcomes (Gasper et al., 2016).

This is the inaugural documentation instance of a proband in a consanguineous family possessing MGVs related to DMD, ODDD, congenital bilateral cataract, and their associated phenotypes.

## Materials and methods

### Ethical permissions

Institutional ethical clearance was obtained from all the participating institutions, and the methods were carried out in accordance with approved guidelines. Written informed consent was obtained for research and publication from the participating family.

### Sample collection and processing

Peripheral blood (5 mL) was obtained from the family member (father, mother, sibling, and proband) in K_2_EDTA vials. Genomic DNA was extracted from whole blood using a rapid non-enzymatic salting-out method (Lahiri et al., 1992), followed by column-based purification using the QIAGEN kit. DNA quality and concentration were assessed using a Qubit fluorometer (Thermo Fisher Scientific).

### Whole-exome sequencing analysis

The purified DNA was subsequently used for whole-exome library preparation with the Twist Exome 2.0 + comp. exome spike-in kit (105035, Twist Bioscience), following the manufacturer’s protocol. WES was performed on the NovaSeq 6000 system (Illumina). Sequencing raw data were obtained for analysis. Raw sequencing data (FASTQ files) underwent quality control using FastQC v0.12.1 and were summarized using MultiQC v1.30. Adapter sequences and low-quality bases were trimmed using Cutadapt v5.1 and Trimmomatic v0.39. High-quality reads were aligned to the human reference genome (GRCh38) using BWA-MEM v0.7.19-r1273. The resulting SAM files were processed with SAMtools v0.1.19, and duplicates were marked using Picard v3.4.0. Variant calling followed the GATK v4.6.2.0 Best Practices workflow, including HaplotypeCaller, base quality score recalibration (BQSR), and joint genotyping. Variants were filtered using standard GATK hard-filtering criteria and annotated with ANNOVAR (2025-03-02 release) and VEP v114, integrating population (gnomAD), functional (SIFT, PolyPhen, CADD, and REVEL), and clinical (ClinVar and OMIM) databases. High-confidence variants (MAF ≤1%) were prioritized based on predicted pathogenicity, inheritance model, zygosity, and Human Phenotype Ontology (HPO) terms. Coverage statistics were assessed using Mosdepth v0.3.11, and variant interpretation and classification were supported by Franklin by Genoox, following the ACMG guidelines. Primer sequences used for PCR amplification and Sanger sequencing validation of the three causal variants were designed to enable family-based segregation analysis (Supplementary Table S1).

### Structural effect of the causal genetic variant

The full-length structures of the wild-type proteins were obtained from the AlphaFold Protein Structure Database (https://alphafold.ebi.ac.uk) using sequences retrieved from the NCBI database (https://www.ncbi.nlm.nih.gov/). Site-directed mutagenesis and truncation of the wild-type proteins were performed using PyMOL 3.1.0 open-source software, and the resulting structures were visualized with UCSF Chimera v1.19.

### Indicative runs of homozygosity and consanguinity analysis

Indicative runs of homozygosity (ROHs) were identified in the proband using autosomal SNP genotype data from WES and analyzed using PLINK v1.9 (‘--homozyg’). ROH were defined as ≥2 Mb regions containing ≥20 consecutive SNPs (≥1 SNP/500 kb), allowing gaps of up to 2 Mb. Sliding windows of 20 SNPs permitted ≤3 heterozygous and ≤5 missing calls, with a homozygosity threshold of 0.05. The final ROH analysis result was visualized using the karyoploteR Bioconductor package.

Autozygosity was assessed through indicative ROH analysis of exome sequencing data. The Twist Human Core Exome BED file was filtered for autosomes (chr1–22) and merged to obtain 35.95 Mb of target regions used for FROH calculation. Indicative ROHs were identified from the filtered autosomal VCF using PLINK, retaining segments that were ≥2 Mb. Intersecting these with the Twist capture regions using Bedtools yielded a total ROH length of 1,423,032 bp (∼1.42 Mb) for the proband.

The fraction of runs of homozygosity (FROH) was calculated using the formula that was logically appropriate for WES data:

$$F_{ROH}=\frac{\text{Total}\;\text{ROH}\;\text{length}\;\text{within}\;\text{capture}}{\text{Total}\;\text{autosomal}\;\text{capture}\;\text{length}}$$

## Results

### Case presentation

**FIGURE 1:**
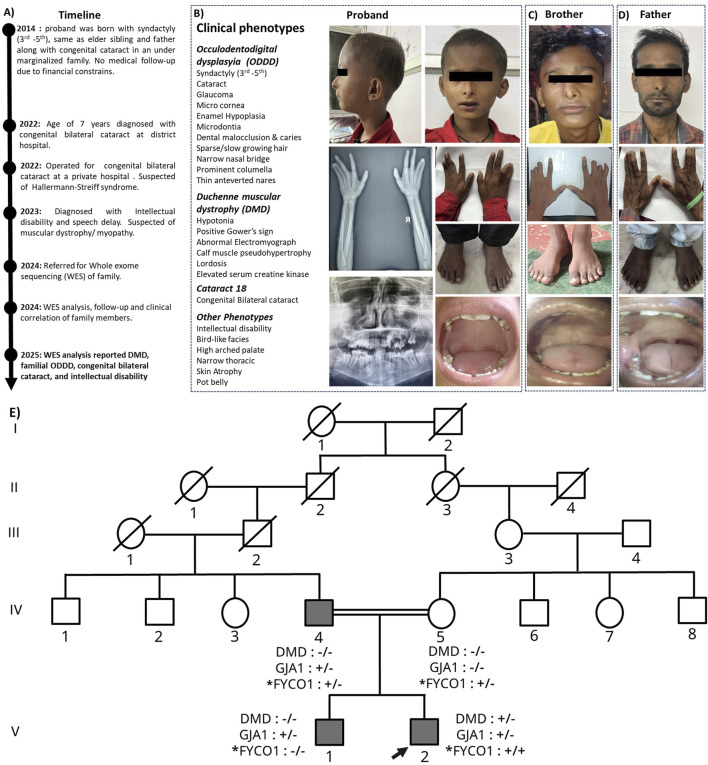
Overview of the proband and the affected members. **(A)** Timeline of clinical findings of the proband. **(B)** Proband and the associated clinical phenotypes. **(C,D)** Affected family members with some shared phenotypes. **(E)** Pedigree of the oculodentodigital dysplasia (ODDD) family. N.B.: “+” indicates the presence of the causal variant, while “–“ indicates its absence; “*” indicates a novel variant; the arrow indicates the proband.

The proband, born in 2014 to an under-marginalized family, presented at birth with syndactyly of the third–fifth digits, similar to the sibling (brother) and father, along with congenital cataracts. Early medical follow-up was not pursued due to financial constraints. At 7 years of age (2022), the proband was diagnosed with congenital bilateral cataracts at a district hospital and later underwent corrective surgery at a private facility, where Hallermann–Streiff syndrome was provisionally considered. In 2023, the proband was identified with intellectual disability and speech delay, raising suspicion of an underlying muscular dystrophy/myopathy. Retrospective parental recalls suggested gait abnormalities and a positive Gowers’ sign beginning at 6–7 years of age, although clinical evaluation was first sought in 2023. Subsequently, routine health checkups and interventions were performed at Shrirama Chandra Bhanja (SCB) Medical College and Hospital, Cuttack, and All India Institute of Medical Sciences, Bhubaneswar. In 2024, the proband and family members were referred for WES for clinical correlation and genetic counseling. Clinical phenotypes of the patient and the family history are summarized in Figure 1. Phenotypes correlating with muscular dystrophy were not observed in any other members of the family (IV:4, IV:5, and V:1).

### Clinical investigations, findings, and patient care

The patient underwent a series of clinical, radiological, and biochemical assessments, the results of which are detailed below.

- *Ultrasonography (USG):* USG of the abdomen and pelvis revealed normal abdominal organs, prostate, and spleen. The left and right kidneys measured 7.4 × 3.2 cm and 7.5 × 3.8 cm, respectively, with normal cortical echotexture and corticomedullary differentiation. Multiple sub-centimeter mesenteric lymph nodes with fatty hilum were noted due to acute gastrointestinal infection.
- *Electromyography (EMG)*: EMG demonstrated myopathic patterns in the examined muscles.
- *Dual-energy X-ray absorptiometry (DEXA):* DEXA revealed normal bone mineral density
- *X-ray of both hands:* The X-ray showed bilateral fusion of the distal phalanges/ syndactyly (third–fifth).
- *Serological analysis:* The level of serum creatine kinase (CK) was 3870 U/L (reference range for male individuals <190; assay method: Rosalski, other modified IFCC).
- *Dental examination:* It revealed enamel hypoplasia, microdontia, dental malocclusions/ caries, and a high arch palate and recommended full-mouth rehabilitation.
- *Psychological evaluation*, *physiotherapist*, *and audiologist/ speech and language therapist:* The results demonstrated intellectual disability with speech delay and slurred speech.
- *Hematological analysis:* The results showed that the complete blood count was within normal/tolerant limits.
- *Genetic diagnosis:* The WES of the family revealed the causal variants that correlated precisely with the clinical phenotypes of the proband and other family members.

#### Patient care

In the late ambulatory phase of DMD, the child received targeted physical and occupational therapy to prolong ambulation, delay contractures, and preserve functional independence. Therapy included daily stretching, gait and balance training, and ankle–foot orthoses/night splints while avoiding high-resistance and eccentric exercises, along with occupational interventions to maintain upper-limb function, support activities of daily living and school participation, and anticipate counseled assistive device needs. After 3–6 months of professionally guided therapy, the parents were trained to continue home-based care and to ensure psychosocial preparedness for the non-ambulatory phase. Pharmacological management included prescribed daily oral corticosteroid therapy and vitamin D and calcium supplementation. Regular cardiac and respiratory surveillance was advised in line with care recommendations to enable early detection of cardiopulmonary complications and optimize long-term outcomes.

### Genetic analysis

#### Multiple genetic diagnoses of multi-locus disease-causing genomic variations

WES and genetic analysis of the proband and the family, performed at the Institute of Life Sciences, Bhubaneswar, identified MGVs that were consistent with MGDs. The analysis revealed disease-causing variants in DMD, GJA1, and FYCO1, collectively explaining the proband’s composite clinical phenotype (Table 1).

**TABLE 1:** Identified multilocus disease-causing genomic variations (MGVs) corresponding to multiple genetic diagnoses (MGDs).

Gene ID	Variant (cDNA; protein notation	Zygosity	Associated disorder	OMIM ID	ACMG evidence codes	Predicted NMD^b^	ACMG-based classification
*DMD*	c.5124_5127del p.Lys1708Asnfs*12	Hemizygous	Duchenne muscular dystrophy	310200	PVS1, PS2, and PM2	Yes	Pathogenic
*GJA1*	c.396_398del p.Ile132_Lys133delinsMet	Heterozygous	Oculodentodigital dysplasia	164200	PM6, PM2, PM4, and PP1	Unmet	Likely pathogenic
*FYCO1* ^a^	c.2482C>T p.Gln828*	Homozygous	Cataract 18	610019	PVS1 and PM2	Yes	Likely pathogenic

^a^ *Novel variant.*

^b^ NMD, nonsense-mediated mRNA decay.

Analysis revealed a comparable variant profile among all family members, with the proband exhibiting the highest variant count (2,322 variants across 1,544 genes). Most variants were benign or likely benign, whereas a small subset (<0.5%) was classified as pathogenic or likely pathogenic, consistent with multi-locus disease-causing genomic variations (MGVs) underlying MGDs. Specifically, a *de novo* hemizygous frameshift variant in DMD (c.5124_5127del; p.Lys1708Asnfs*12; NM_004006.3) was associated with DMD (OMIM: 310200), a heterozygous in-frame indel in GJA1 (c.396_398del; p.Ile132_Lys133delinsMet; NM_000165.5) was linked to ODDD (OMIM: 164200), and a novel homozygous nonsense variant in FYCO1 (c.2482C>T; p.Gln828*; NM_024513.4) accounted for congenital cataract/ cataract 18 (OMIM: 610019). These findings underscore multi-locus pathogenicity in a consanguineous family presenting with overlapping neuromuscular, craniofacial, and ocular phenotypes. A summary of variant counts, filtration strategy, and phenotype-based (HPO) filtering parameters used in Franklin (www.franklin.genoox.com) for family WES analysis is detailed in Supplementary Table S2.

### Structural effect of the causal genetic variants

**FIGURE 2:**
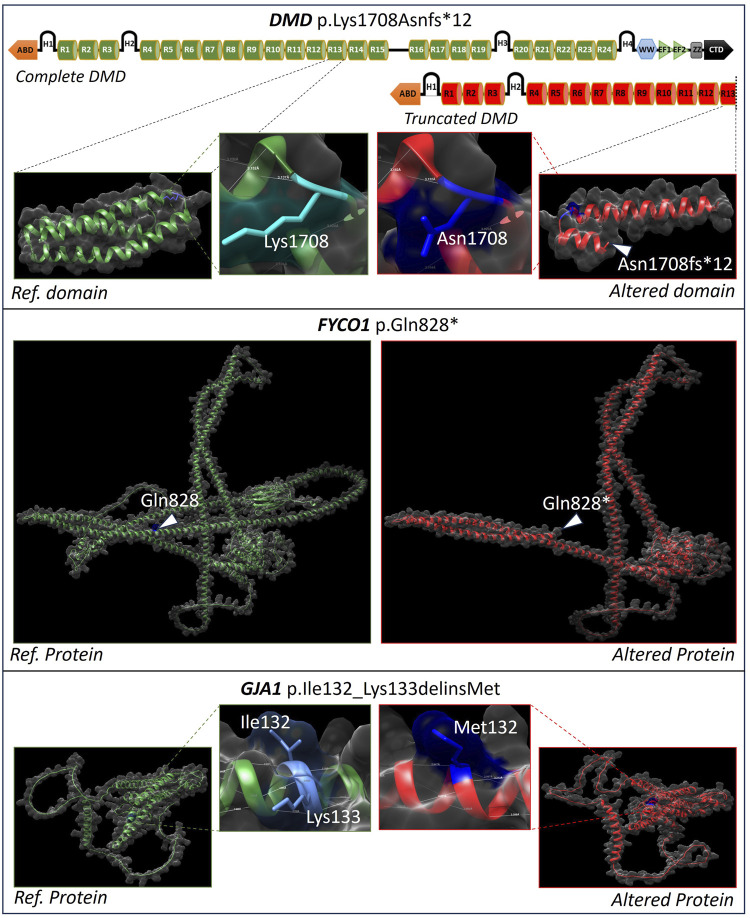
Effect of causal variants on the protein structure. Premature truncation of dystrophin (*DMD:* p.Lys1708Asnfs*12) and FYCO1 (p.Gln828*), and a local in-frame indel in connexin 43 (*GJA1*: p.Ile132_Lys133delinsMet) affecting protein conformation and functional domains.

Two of the identified variants, DMD: p.Lys1708Asnfs*12 and FYCO1: p.Gln828*, result in premature protein truncation. The DMD frameshift causes early termination beyond leucine 1720, eliminating key dystrophin domains. The FYCO1 nonsense variant truncates the autophagy adapter protein at residue 828, leading to the loss of the FYVE, LIR, and GOLD domains, which are essential for autophagic transport. In contrast, the GJA1 variant (p.Ile132_Lys133delinsMet) represents a small in-frame indel within a surface-exposed, moderately conserved region of connexin 43, potentially altering gap junction assembly or gating. The structural impact of these causal variants, depicting changes from the reference to the altered protein conformation, is illustrated in Figure 2.

### Family history and pedigree analysis

Analysis of the family revealed distinct inheritance patterns for the causal variants. The DMD variant (p.Lys1708Asnfs*12) is hemizygous in the proband and occurred *de novo* as it was absent in both parents. The GJA1 variant (p.Ile132_Lys133delinsMet) is heterozygous, inherited from the father, and is also present in the brother (autosomal dominant). The FYCO1 variant (p.Gln828*) is homozygous in the proband, with both parents as heterozygous carriers (autosomal recessive). These inheritance patterns are clearly illustrated in Figure 1E and Supplementary Figure S1.

### Indicative runs of homozygosity and consanguinity validation by FROH

**FIGURE 3:**
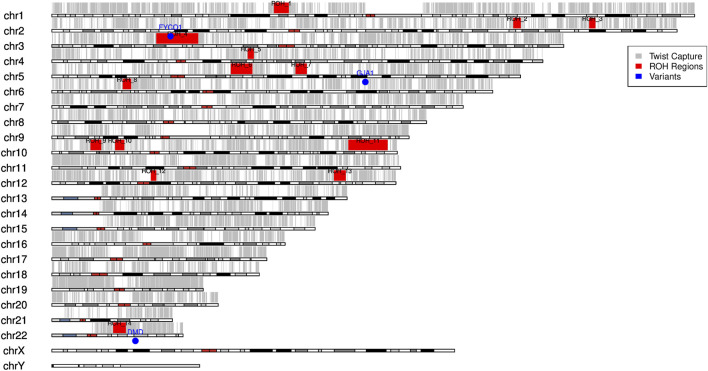
Indicative PLINK–ROHs of the proband. Summary of ROHs identified in the proband from whole exome data using PLINK within the exome capture regions. A total of 14 ROHs were identified across nine autosomes. ROH regions (shown in red) begin on chromosome 1 (ROH1) and are sequentially numbered across the autosomes, continuing up to ROH14 on chromosome 22. Causal variants are marked with a blue label/dots.

An indicative trend of ROHs was identified for the proband from WES data using PLINK, with a minimum threshold of 2 Mb to capture extended autozygous regions (Figure 3). Details of the indicative PLINK–ROH coordinates and metrics are provided in Supplementary Table S3.

For the assessment of potential parental relatedness (consanguinity), we followed ACMG recommendations and emphasized longer indicative ROHs. Specifically, only ROHs ≥10 Mb and present on multiple autosomes were considered significant as such segments are unlikely to occur by chance or due to linkage disequilibrium and are widely accepted indicators of recent parental relatedness. Shorter ROHs (5 Mb–10 Mb) were noted but interpreted with caution, given that they may reflect background population structure, particularly in exome-based analysis, where coverage is not genome-wide. A causal variant (homozygous *FYCO1* c.2482C>T on chr 3 within ROH4) lies within an indicative homozygous tract, indicating identity-by-descent (IBD). Autozygosity was assessed from exome sequencing, identifying 1.42 Mb of ROHs in 35.95 Mb of autosomal capture regions for the proband, which was used for FROH calculation (Table 2).

**TABLE 2:** Summary for the indicative ROHs and FROH.

ID	Total ROH in capture (Mb)	FROH (%)
Proband	1.42	0.0396 (3.96%)

The pedigree and FROH analyses indicate possible parental consanguinity, consistent with a relationship equivalent to second cousins or closer. This interpretation is supported by the presence of long contiguous PLINK indicative ROHs (up to 15 and 16 Mb in the proband) and several additional indicative tracts exceeding 5 Mb, which may signify recent shared ancestry. Together, the inheritance pattern and exome-derived FROH findings highlight multi-locus involvement within this consanguineous family.

## Discussion

The *FYCO1* (FYVE and Coiled-Coil Domain Containing 1) gene, located on chromosome 3p21.31, encodes an autophagy adapter protein that interacts with LC3 (microtubule-associated protein 1A/1B-light chain 3) and Rab7, a small GTPase. Through these interactions, *FYCO1* mediates the microtubule-dependent transport of autophagosomes toward the cell periphery, thereby facilitating the degradation of damaged α-crystallin proteins—major structural components that are essential for ocular lens transparency (Chen et al., 2011; Olsvik et al., 2015).

The identified variant, FYCO1:c.2482C>T (p.Gln828*), is located in exon 8 of the transcript NM_024513.4. This single-nucleotide substitution (C>T) at coding position 2,482 changes the codon for glutamine (CAG) to a premature stop codon (TAG), resulting in a nonsense (stop-gain) mutation. The alteration is predicted to generate a truncated protein lacking the downstream coiled-coil and FYVE domains that are crucial for vesicular trafficking or to trigger nonsense-mediated mRNA decay (NMD), leading to the complete loss of protein function and potentially causing autosomal recessive congenital cataract (OMIM #610019), which is characterized by early-onset bilateral lens opacity typically without systemic involvement. Accordingly, the FYCO1 variant is interpreted as likely pathogenic, consistent with the established disease mechanism. The FYCO1:c.2482C>T variant was absent from major population databases (gnomAD, ExAC, and IndiGenomes) and clinical databases (ClinVar and LOVD). Additionally, no entries were found in PubMed, Google Scholar, or the Franklin (Genoox) database; the variant was also absent from other population datasets (1000 Genomes, ESP6500, ExAC, and UK10K), indicating that it is a novel variant.

While cataracts have occasionally been reported in patients with ODDD (Kumar et al., 2020), the congenital bilateral cataract observed in the proband of the present study is most likely attributable to the novel FYCO1 variant. The likely pathogenic GJA1 variant, which is responsible for ODDD, is also present in the father and brother, neither of whom exhibits a cataract phenotype, supporting the independent contribution of the FYCO1 variant to the ocular manifestation.

The DMD: c.5124_5127del (p.Lys1708Asnfs*12; rs398123979) variant results in a frameshift and premature termination, leading to dystrophin loss of function. It fulfills PVS1 (predicted loss of function) and is classified as pathogenic according to the ACMG guidelines. This frameshift disrupts the open reading frame, resulting in a truncated dystrophin protein lacking essential functional domains required for normal muscle function, as observed in the proband. This variant represents a null allele in DMD, a gene with a well-established loss-of-function disease mechanism, supported by 1,841 pathogenic truncating variants reported in ClinVar across 77 exons (including 27 in exon 36) and strong loss of function (LoF) constraint (gnomAD observed/expected = 0.154). The variant affects coding exon 36/79 and is located upstream of the terminal exon, not within the last 50 bp of the penultimate exon, and is, therefore, predicted to undergo nonsense-mediated mRNA decay.

The DMD frameshift indel c.5124_5127del is recorded as pathogenic in clinical databases (ClinVar) with two independent submissions linking it to DMD, but no peer-reviewed case reports providing detailed genotype–phenotype correlation for this exact variant are currently available in accessible literature. Parental and sibling testing by WES with orthogonal Sanger sequencing did not detect the DMD variant, which is consistent with a *de novo* event. However, ultra-low level maternal mosaicism cannot be excluded as the currently used methodologies are not optimized for the reliable detection of low-variant allele fraction events and would require ultra-deep targeted sequencing for definitive exclusion.

Variations in GJA1 (connexin 43) act by disrupting gap junction communication through several mechanisms, including protein mislocalization, failure to form connexons, or altered channel conductance and gating. Many ODDD-associated missense variants cluster in a region homologous to the calmodulin-binding motif, affecting calcium-dependent regulation. Functional studies of related connexins indicate that these mutations often act in a dominant-negative manner, where mutant subunits interfere with wild-type connexin function during embryonic development (Paznekas et al., 2003; Sargiannidou et al., 2021). The GJA1:c.396_398del (p.Ile132_Lys133delinsMet; rs1773903559) variant, identified in ClinVar but not specifically linked to the ODDD phenotype, represents an in-frame deletion altering conserved residues in connexin 43. Based on the combined ACMG criteria codes, it is classified as likely pathogenic.

None of the three causal variants were detected in major population databases such as gnomAD or ExAC, highlighting their rarity. Similarly, Franklin (Genoox) searches showed no entries across other population datasets (1000 Genomes, ESP6500, ExAC, and UK10K), confirming their absence in global cohorts.

PLINK-based indicative ROHs and FROH analyses from exome data indicate extended autozygosity (up to 15 Mb–16 Mb), which is consistent with parental consanguinity equivalent to second cousins or closer. The novel homozygous FYCO1 variant was localized within one such ROH, indicating identity-by-descent, while the DMD variant arose *de novo*. The coexistence of recessive, dominant, and *de novo* variants highlights the diagnostic complexity of multi-locus pathogenicity in consanguineous families.

The presence of the mentioned three pathogenic or likely pathogenic variants within a single individual is extremely rare and a first report. Each gene is independently associated with a distinct Mendelian disorder: FYCO1 with autosomal recessive congenital cataract, DMD with *de novo* appearance, and GJA1 with autosomal dominant oculodentodigital dysplasia. This exceptional co-occurrence underscores the importance of comprehensive genomic analysis for accurate diagnosis and phenotypic correlation. Such multi-locus variant combinations are scarcely reported and emphasize the contribution of MGVs to phenotypic diversity and variable expressivity in Mendelian disorders.

The relevance of this type of case lies primarily in the diagnostic, interpretative, and counseling challenges it presents rather than its frequency. In patients with complex or multisystem phenotypes, the pursuit of a single unifying molecular diagnosis may lead to diagnostic anchoring, particularly when some clinical features are discordant or exceed the known spectrum of a single disorder. In this case, systematic phenotypic stratification and independent evaluation of major clinical manifestations enabled more accurate attribution of the findings to distinct genetic etiologies.

From a medical genetics perspective, the coexistence of multiple molecular diagnoses necessitates independent interpretation of each variant with respect to the inheritance pattern, prognosis, and recurrence risk, thereby complicating genetic counseling and family risk assessment. Furthermore, this study highlights the importance of distinguishing true phenotypic expansion of a genetic disorder from the coexistence of multiple molecular defects as apparently broadened phenotypes may be more parsimoniously explained by separate genetic conditions.

In some complex cases, additional diagnoses may escape standard NGS pipelines due to technical or interpretative limitations, thus underscoring the value of careful phenotyping, periodic data reanalysis, and close clinician–geneticist collaboration in the evaluation of complex cases.

The present study exemplifies the clinical and genetic complexity arising from multi-locus variation in a consanguineous pedigree and highlights the importance of trio/family-based exome sequencing for accurate diagnosis, genetic counseling, and phenotype interpretation in rare genetic disorders.

## Data Availability

The data that support the findings of this study are available on request from the corresponding author. The data are not publicly available due to privacy or ethical restrictions.
