# Supplementary material for: Co-occurring DMD, GJA1, and novel FYCO1 variants in a proband from a consanguineous oculodentodigital dysplasia family: a rare multi-locus case report
Source: Front Genet. 2026 Mar 4;17:1753212. doi: 10.3389/fgene.2026.1753212 (PMC12995185; doi:10.3389/fgene.2026.1753212)
Supplement: Supplementary file 1 [file DataSheet1.docx]

**SUPPLEMENTARY MATERIALS**

**Supplementary Table S1|** Primer sequences used for PCR amplification and Sanger sequencing validation of the three causal variants for family-based segregation analysis.

| **Gene** | **Causal Variant** | **Primer Sequence 5’ to 3’** | **Annealing temperature** | **Amplicon size (in bps)** |
| --- | --- | --- | --- | --- |
| ***DMD*** | c.5124_5127del | F: ACACCCTTCTCTGTCACGAG | 57 ^0^C | 580 bps |
|  |  | R: TCATTCATTTCCATTCAAAGGGG |  |  |
| ***FYCO1*** | c.396_398del | F: GGGGGAGGTCCAACGGCTGC | 65 ^0^C | 304 bps |
|  |  | R: GCTGCATTTGGCCTGGGACA |  |  |
| ***GJA1*** | c.2482C>T | F: TGTACCTGGCTCATGTGTTCT | 52.5 ^0^C | 248 bps |
|  |  | R: CTGGATCAGCAAGAAGGCCA |  |  |

**Supplementary Table S2|** Number/status of variants, variant filtration criteria, and HPO terms used for phenotype-based filtering in Franklin during the family’s whole exome analysis.

| **Variants/Status** | **Proband** | | **Brother** | | | **Father** | | | | **Mother** | | | |
| --- | --- | --- | --- | --- | --- | --- | --- | --- | --- | --- | --- | --- | --- |
|  | **Variants** | **Genes** | **Variants** | **Genes** | | **Variants** | | **Genes** | | **Variants** | | **Genes** | |
| **VCF filtered Total** | 31262 | 11183 | 28124 | 10558 | | 28065 | | 10619 | | 27944 | | 10654 | |
| **Franklin filtered Total** | 2322 | 1544 | 1835 | 1341 | | 1817 | | 1328 | | 1779 | | 1323 | |
| **Benign** | 992 | 818 | 878 | 719 | | 851 | | 723 | | 818 | | 703 | |
| **Likely Benign** | 287 | 242 | 238 | 203 | | 213 | | 183 | | 235 | | 201 | |
| **VUS** | 1034 | 582 | 708 | 486 | | 743 | | 505 | | 717 | | 501 | |
| **Likely Pathogenic** | 4 | 5 | 7 | 7 | | 7 | | 7 | | 7 | | 7 | |
| **Pathogenic** | 5 | 5 | 4 | 4 | | 3 | | 3 | | 2 | | 2 | |
| **Identified Causal variant post phenotype corelation** | 3 | 3 | 1 | 1 | | 1 | | 1 | | 0 | | 0 | |
| **Phenotype/Clinical/HPO terms used for variant identification** | | | | | | | | | | | | | |
|  | HP:0000218 | | HP:0010710 | | | HP:0010710 | | | | NA | | | |
|  | HP:0000320 | | HP:0000486 | | | HP:0000486 | | | | NA | | | |
|  | HP:0000369 | |  |  | |  | |  | |  | |  | |
|  | HP:0000446 | |  |  | |  | |  | |  | |  | |
|  | HP:0000486 | |  |  | |  | |  | |  | |  | |
|  | HP:0000519 | |  |  | |  | |  | |  | |  | |
|  | HP:0000582 | |  |  | |  | |  | |  | |  | |
|  | HP:0000750 | |  |  | |  | |  | |  | |  | |
|  | HP:0000750 | |  |  | |  | |  | |  | |  | |
|  | HP:0001591 | |  |  | |  | |  | |  | |  | |
|  | HP:0001999 | |  |  | |  | |  | |  | |  | |
|  | HP:0003236 | |  |  | |  | |  | |  | |  | |
|  | HP:0003307 | |  |  | |  | |  | |  | |  | |
|  | HP:0003307 | |  |  | |  | |  | |  | |  | |
|  | HP:0003391 | |  |  | |  | |  | |  | |  | |
|  | HP:0003552 | |  |  | |  | |  | |  | |  | |
|  | HP:0003698 | |  |  | |  | |  | |  | |  | |
|  | HP:0008070 | |  |  | |  | |  | |  | |  | |
|  | HP:0008981 | |  |  | |  | |  | |  | |  | |
|  | HP:0009118 | |  |  | |  | |  | |  | |  | |
|  | HP:0009765 | |  |  |  | |  | |  | |  | |  |
|  | HP:0010710 | |  |  | |  | |  | |  | |  | |

**Supplementary Table S3|** Summary of indicative runs of homozygosity (ROH) identified in the proband from whole exome data using PLINK.

| **Chr No.** | **ROH Start** | **ROH end** | **ROH in KB** | **nSNP** | **DENSITY** | **PHOM** | **PHET** |
| --- | --- | --- | --- | --- | --- | --- | --- |
| 1 | 86022549 | 91797317 | 5774.8 | 39 | 148.071 | 0.846 | 0.154 |
| 2 | 178750225 | 181678728 | 2928.5 | 25 | 117.14 | 0.88 | 0.12 |
| 2 | 208121913 | 210616533 | 2494.6 | 24 | 103.943 | 0.875 | 0.125 |
| 3 | 40462119 | 56729300 | 16267.2 | 221 | 73.607 | 0.964 | 0.036 |
| 4 | 75866520 | 78265017 | 2398.5 | 22 | 109.023 | 0.864 | 0.136 |
| 5 | 69320504 | 77715241 | 8394.7 | 42 | 199.875 | 0.857 | 0.143 |
| 5 | 94386345 | 98780124 | 4393.8 | 33 | 133.145 | 0.97 | 0.03 |
| 6 | 27457406 | 30744028 | 3286.6 | 93 | 35.34 | 0.914 | 0.086 |
| 10 | 14934415 | 19136775 | 4202.4 | 31 | 135.56 | 0.806 | 0.194 |
| 10 | 24501464 | 28089829 | 3588.4 | 33 | 108.738 | 0.848 | 0.152 |
| 10 | 114833416 | 130079827 | 15246.4 | 113 | 134.924 | 0.965 | 0.035 |
| 12 | 38318340 | 40486507 | 2168.2 | 50 | 43.363 | 0.9 | 0.1 |
| 12 | 109285272 | 113858782 | 4573.5 | 34 | 134.515 | 0.824 | 0.176 |
| 22 | 23743920 | 28679646 | 4935.7 | 40 | 123.393 | 0.85 | 0.15 |

Total 14 indicative PLINK ROHs across 9 autosomes were identified for suggestive autozygosity.

**Supplementary Figure**

**
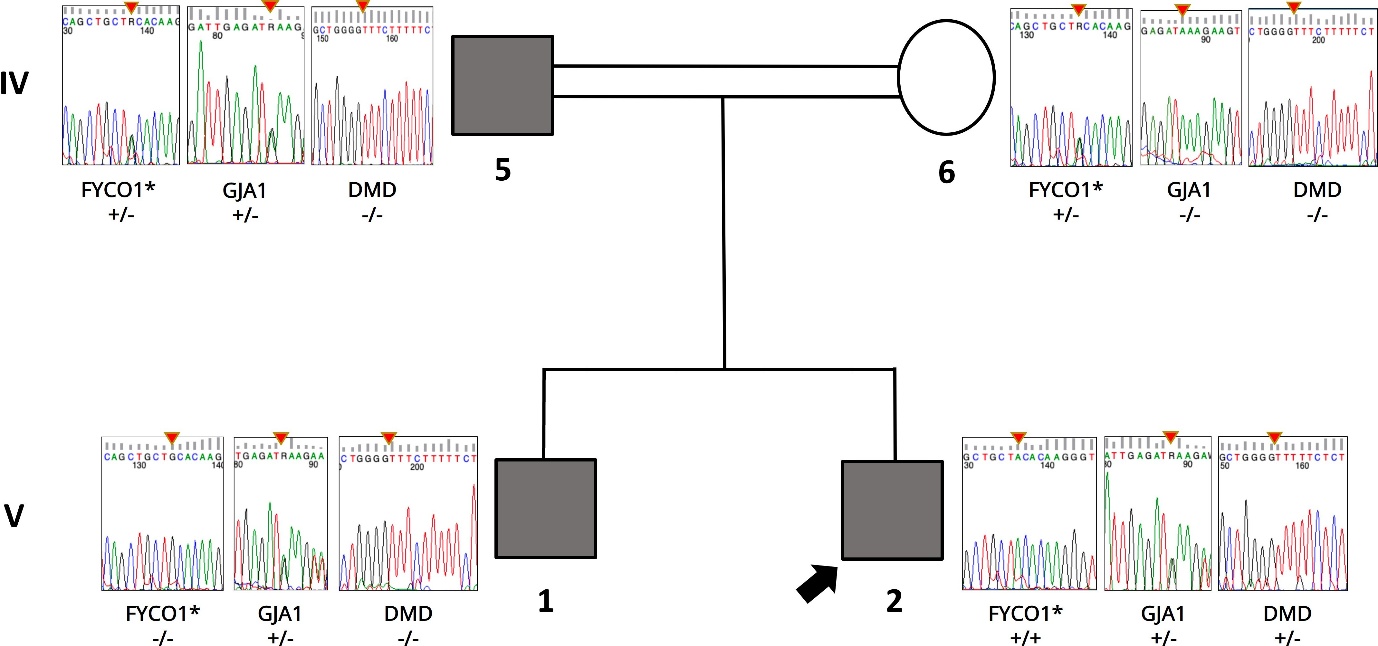
**

**Supplementary Figure S1|** Pedigree illustrating segregation of three causal variants validated by Sanger sequencing. Representative Sanger chromatograms and corresponding genotype statuses (zygosity) are shown. “+” indicates the presence and “–” indicates the absence of the causal variant; “*” denotes a novel variant; arrows indicate the proband.
